# Internal Control Quality, Enterprise Environmental Protection Investment and Finance Performance: An Empirical Study of China’s A-Share Heavy Pollution Industry

**DOI:** 10.3390/ijerph17176082

**Published:** 2020-08-21

**Authors:** Liu Yang, Han Qin, Quanxin Gan, Jiafu Su

**Affiliations:** 1 School of International Education, Guangxi University of Finance and Economics, Nanning 530003, China; liuyang_vinda@hotmail.com; 2 MPAcc Center, Guangxi University of Finance and Economics, Nanning 530003, China; xxsqinhan@hotmail.com; 3 Admissions and Employment Office, Guangxi University of Finance and Economics, Nanning 530003, China; 4 International College, National Institute of Development Administration, Bangkok 10240, Thailand; 5 National Research Base of Intelligent Manufacturing Service, Chongqing Technology and Business University, Chongqing 400067, China

**Keywords:** environmental protection investment, internal control, financial performance, heavy pollution industry, environmental sustainability

## Abstract

As an important measure of enterprise governance, internal control can enhance the organizational rationality of the enterprise, ensure that the enterprise consciously assumes social responsibility for the protection of the natural environment and resources, and promote the sustainable development of the national economy. Using data from China’s A-share heavy pollution industry listed companies from 2009 to 2018, this study explored the relationships among internal control quality, enterprise environmental protection investment, and financial performance. The results show that the quality of internal control has a significant positive impact on enterprise environmental protection investment and financial performance. Enterprise environmental protection investment has a significant positive impact on financial performance and plays a partial intermediary role in the positive impact of internal control quality on financial performance. While expanding the theory of resource-based concepts, this study clarified the positive impact of corporate environmental management and practical behavior on corporate value and provides a theoretical basis for companies to actively implement environmental protection responsibilities, strengthen internal environmental management capabilities, and enhance corporate value. At the same time, it also provides a basis for the government to issue relevant environmental protection policies, strengthen enterprise internal control construction guidelines, and encourage third-party organizations to evaluate the effectiveness of enterprise internal control.

## 1. Introduction

Against the backdrop of the new economic normal, China continues to promote structural reforms on the supply side, strengthen environmental constraints, and strictly control environmental protection energy consumption. As the strategic position of ecological environmental protection continues to improve, the concept of green development has become a general consensus in society. As a cell of the national economy, enterprises are direct bearers of social production and circulation. At the same time, it is also a major consumer of resources and a manufacturer of environmental pollution. It has an inescapable responsibility for environmental protection. However, even with the increasingly stringent external environmental regulations, Chinese enterprises still lack the enthusiasm for environmental protection investment. This is mainly due to the characteristics of large investment, long cycle, and the lack of explicit investment income in environmental protection investment activities, which further limits the sustainable development of enterprises and the improvement of environmental performance. Therefore, there is an urgent need to explore the resolution mechanism from the internal perspective of the enterprise itself.

On the one hand, internal control, as an important way of corporate governance, not only profoundly affects the operation and management of the enterprise, but also leads in resource allocation and other aspects, and it has been proven to have a positive effect on the financial performance of the enterprise. Many studies have shown that: First, enterprises with internal control defects will increase the risk of falling stock prices [1], leading to higher financial risks [2] and cost of equity capital [3]. The higher is the cost of equity capital, the higher are the requirements of the shareholders and creditors for the enterprise, which will increase the debt repayment risk of the enterprise and the investment risk of subsequent operations, reduce the financing space, inhibit the value increase of the enterprise, and thus have a negative impact on the financial performance of the enterprise [4]. Second, the stronger is the internal control effectiveness (quality), the more it can improve the quality of accounting information and earnings [5]. At the same time, the more comprehensive is the internal control information disclosure, the higher are the manager’s confidence in decision-making and the legal compliance of the company’s business performance [6,7]. It can create good business environment for the company [8], which is conducive to the improvement of the company’s financial performance, and its positive impact is positively demonstrated.

On the other hand, internal control will promote corporate social responsibility and corporate environmental performance. It has a certain spillover effect. In the early stage, many studies have revealed the important impact of internal governance structure on the quality of corporate environmental information disclosure and environmental protection practices [9] from management characteristics [10], shareholding concentration, board structure [11], etc. It also reflects the promotion of internal control quality’s effectiveness to enterprise environmental protection practices. However, there is still little research on the relationship between internal control quality and enterprise environmental protection investment.

At present, driven by the economic motive for profit maximization, enterprises are still passively performing environmental responsibilities under the compulsory constraints of strict laws and regulations. Based on this, this study started from the perspective of enterprise management capabilities and explored the path to achieve the “win–win” goal of corporate environmental performance and financial performance under the intermediary transmission of enterprise environmental protection investment. In addition, existing research in China mainly uses the nature of enterprise property rights [12] and shareholding structure [13] as the moderating variables of the relationship between internal control quality and enterprise performance, or uses the enterprise social responsibility [14], agency costs [15], and overconfidence of management [16] as the intermediary variables. The above studies lack the practical needs based on the construction of ecological civilization and fail to organically link the three relationships by using enterprise environmental protection investment as an intermediary variable. Therefore, this study used enterprise environmental protection investment as an intermediary variable with reasonable internal logic and realistic background.

## 2. Theoretical Background and Hypotheses

### 2.1. The Impact of Internal Control Quality on Financial Performance

With the development of productive forces and the continuous refinement of the social division of labor, the principal–agent relationship has become the basic relationship of enterprise internal governance. However, asset owners (principals) are often at a disadvantage in terms of access to information, while specific managers (agents) may invade the interests of the enterprise based on maximizing private interests and affect the rights of shareholders and other stakeholders. It leads to agency cost problems and inhibits the efficiency of internal capital development.

As a management method, internal control is guided by value creation to promote economic growth and obtain a higher return on investment [17], which is used to improve operations and transform financial policies. In essence, internal control is a series of institutional policies and procedural measures related to the control environment, information and communication, risk assessment, control measures, and supervisory feedback for all members, including business owners and managers [18]. On the one hand, the information disclosed by the enterprise is the basis for investors and other stakeholders to make judgments on the operation of the enterprise, and it is also the key to building stakeholder trust. The fifth chapter of China’s “The basic norms of internal control” clearly requires enterprises to establish information and communication systems to improve the usefulness of information. Therefore, improving the quality of internal control is conducive to reducing the degree of asymmetry of internal and external information, and enhancing the trust of stakeholders, so that they can comprehensively understand the real operating conditions and development capabilities of the enterprise. Finally, it will provide guarantee for enterprises to obtain more financial support. On the other hand, in the absence of constraints and reward mechanisms, enterprise managers can easily use company resources to realize their desire for private gain. Using internal control to establish a restraint and punishment mechanism, enterprises will standardize and institutionalize internal governance and management behavior, which can effectively control management’s speculative self-interest, ease internal agency problems, and create a good internal environment for improving corporate financial performance. Based on the above analysis, we propose our first hypothesis:

**Hypothesis** **1** **(H1).** *The quality of internal control has a positive impact on financial performance.*

### 2.2. The Impact of Internal Control Quality on Enterprise Environmental Protection Investment

Enterprise environmental protection investment is the performance of specific practical activities for enterprises to fulfill their social responsibility. When carrying out environmental protection investment activities, enterprises often need a large amount of funds to pay for the high cost of environmental protection equipment and environmental technology innovation. This is a non-economic project that requires long-term investment, and the return of economic benefits in a short time is difficult to achieve [19,20]. Based on neoclassical theory, the enterprise is a for-profit social organization, and the maximization of interest is its goal. Without institutional constraints and economic incentives, enterprises often lack enthusiasm for environmental protection investment activities [21,22,23]. Therefore, to promote enterprises to actively fulfill their environmental responsibilities, in addition to ensuring adequate funding sources, more institutional support is needed [24]. China’s “Internal Control Application Guidelines No. 4: Social Responsibility” states that companies should pay attention to the risk of huge compensation for companies due to insufficient environmental protection investment and large resource consumption. Its Chapter 4 clarifies that companies need to establish environmental protection systems and other specific provisions to promote enterprises to fulfill their environmental responsibility [25]. It not only regulates the enterprise’s decision-making behavior and strengthens the rationality of the enterprise’s organizational structure, but also promotes the enterprise to organically embed the needs of stakeholders and corresponding social responsibilities.

On the other hand, as the specific operator of the enterprise, the management has the characteristic of “limited rationality” in the complex and changing internal and external environment. Because environmental protection investment has the risk of income uncertainty, managers have resistance to environmental protection investment. In terms of environmental protection decisions, the enterprise’s major shareholders and management often appear to “collude” [21]. With the stronger environmental protection demands of stakeholders, enterprises urgently need to improve the supervisory mechanism and incentive mechanism to effectively control internal management’s adverse selection and moral hazard [26]. It reduces the external costs by circumventing “collusion”, promotes enterprise managers to invest more resources in environmental protection projects to meet the expectations of stakeholders under the standardization of corporate environmental responsibility. Long et al. [27] believed that enterprises should respond to the demands of stakeholders and exert the standardization role of internal control in the realization of social responsibility of enterprises, so as to promote managers to invest more resources in social responsibility projects and achieve legality goals. This leads to our second hypothesis:

**Hypothesis** **2** **(H2).** *The quality of internal control has a positive impact on the scale of enterprise environmental protection investment.*

### 2.3. The Impact of Enterprise Environmental Protection Investment on Financial Performance

Enterprise environmental protection investment, similar to traditional investments, follows the basic investment principles. It must not have a negative impact on enterprise value. Although the definition of enterprise environmental protection investment has not been uniformly defined, scholars agree that it is an investment activity with environmental protection as the main purpose and can produce significant environmental performance. However, in terms of its economic performance, the previous studies did not form a unified conclusion. At present, the mainstream view that enterprise environmental protection investment has a positive impact on financial performance is mainly based on the Porter hypothesis and resource-based view. On the one hand, according to the Porter hypothesis, enterprise environmental protection investment will encourage process innovation and product innovation activities. The value-added effect generated by innovation will offset the huge cost of environmental protection investment. Moreover, it will lead to “first mover advantage” and “innovation compensation” by virtue of technological innovation capabilities [28,29]. This will further improve the efficiency of resource utilization, reduce the risk of illegal taxes and fees, and benefit economic efficiency.

On the other hand, the resource-based view also believes that the environmental governance activities paid by enterprises are conducive to enhancing the “heterogeneity” resources of enterprises and exerting their unique competitive advantages [30]. In addition, enterprises environmental protection investment is a concrete manifestation of their active commitment to social responsibility, which can shape a good social image, satisfy stakeholders’ demands for environmental protection; obtain the trust of external investors, suppliers, customers, and other stakeholders in the enterprises and products; and reduce the cost of raw materials, manpower, and services. At the same time, it further enhances the company’s reputation, sales, and equity. It reduces the cost of financing, environmental taxes and fines, and brings a “green premium”. Finally, it creates good conditions for more operating income [31]. Based on this, we propose our third hypothesis:

**Hypothesis** **3** **(H3).** *Enterprise environmental protection investment has a positive impact on financial performance.*

**Figure 1:**
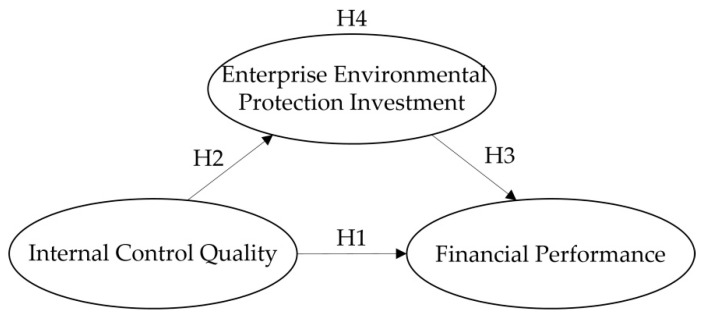
Theoretical model.

In summary, internal control, as an effective mechanism for enterprise governance, not only affects financial performance, but also promotes enterprise social responsibility practices. Good internal control will directly promote the improvement and manifestation of financial performance. At the same time, it will also restrain the “pure profit-seeking” behavior of enterprises and restrict the opportunistic behavior of managers, which enables the effective implementation of environmental protection investment activities, brings green premiums, and further improves financial performance. This shows that enterprise environmental protection investment plays an intermediary role in the process of internal control affecting financial performance. Therefore, we propose the following theoretical model show in Figure 1, as well as the fourth hypothesis:

**Hypothesis** **4** **(H4).** *Enterprise environmental protection investment plays an intermediary role in the impact of internal control quality on financial performance.*

## 3. Methods

### 3.1. Data Sources

In this study, data of listed companies in heavily polluted A-share industries in China from 2009 to 2018 were used as samples, and data samples of ST and * ST companies were deleted, because ST and * ST companies mean that they have been specially processed by the China Securities Exchage and have a greater risk of delisting. Finally, 2326 valid samples were obtained. The enterprise internal control index was selected from the database of DIB internal control and risk management. The scale of enterprise environmental protection investment and other index data were selected from the CSMAR database and obtained by manual sorting.

### 3.2. Measurement of the Main Variables

#### 3.2.1. Explanatory Variable

Internal control quality (ICQ): At present, the internal control index of Chinese listed companies is mainly divided into two versions published by Xiamen University and Dibo Company in Xiamen, China. Compared with the former, Dibo’s internal control index aims to evaluate the achievement of the five major goals of corporate internal control: compliance, reporting, operation, asset safety, and strategy. At the same time, it revises the basic index of internal control by modifying variables (internal control defects) and constructs an evaluation index that integrates internal control objectives, elements, economic effects, and financial data. Therefore, it can more objectively reflect the degree of implementation of the enterprise’s internal control construction [27,32]. In addition, the Dibo’s internal control index is released yearly, which also guarantees the integrity of the time series. Therefore, Dibo’s internal control index has higher reliability and rationality. Following Li and Zhao [33], this study used the natural logarithm of Dibo’s internal control index.

#### 3.2.2. Explained Variable

Financial performance (EPS): In general, financial performance reflects the management efficiency and overall operating conditions of an enterprise within a certain operating period. Measuring financial performance generally includes market indicators and accounting indicators. Considering the influence of market volatility, this study chose the latter to measure financial performance. Among accounting indicators, the most commonly used are return on equity (ROE) and earnings per share (EPS). This study chose EPS as the proxy variable of financial performance [6], mainly because ROE is easily affected by the asset–liability ratio and cannot comprehensively reflect the true financial status of the enterprise. Among the profitability indicators of listed companies, EPS, as one of the most important indicators in financial reports, represents the company’s after-tax profit per share. It is a relatively objective and direct ratio indicator that measures corporate profitability and can better reflect operating conditions.

#### 3.2.3. Intermediary Variable

Enterprise environmental protection investment (EPI): At present, the definition of corporate environmental protection investment in academia has not yet been uniformly defined. Most scholars’ explanations of EPI tend to: For the purpose of preventing pollution and protecting the environment, enterprises achieve special economic activities while taking into account environmental and social benefits [34]. This study used the natural logarithm of the newly added environmental protection investment of the enterprise to measure EPI and effectively reduced the impact of the enterprise scale on it [33,35].

#### 3.2.4. Control Variable

Following the conventional practice of the existing literature [33,34,35,36,37,38], this study considered the internal characteristics of the company and external environmental indicators. The asset–liability ratio (LEV), asset turnover ratio (ATO), proportion of independent directors (ID), operating risk (VOL), CEO change (TURN), institutional investor holdings (INSHARE), enterprise size (SIZE), market the degree of competition (HHI), and annual dummy variables (YEAR) were used as control variables. The variable description is shown in Table 1.

### 3.3. Model Design

This study constructed Models (1)–(3) and used the stepwise regression method to test the intermediary effect of corporate environmental protection investment:

Firstly, examine the impact of internal control quality on financial performance. If *β*_1_ in Model (1) is positive and significant, a basis for further testing of mediation effects is established.

(1) $$\begin{array}{c} EPS=\alpha +\beta_{1}ICQ+\beta_{2}LEV+\beta_{3}ATO+\beta_{4}ID+\beta_{5}VOL+\beta_{6}TURN+\beta_{7}INSHARE\\ +\beta_{8}SIZE+\beta_{9}HHI+\sum YEAR\_Dummy+\varepsilon \end{array}$$

Secondly, test Models (2) and (3) in turn. If *β*_1_ in Model (2) is positive and significant and *β*_2_ in Model (3) is positive and significant, it means that the quality of internal control is a significant way to achieve financial performance through enterprise environmental protection investment.

Finally, make further judgments based on the test results of Model (3). If *β*_1_ is not significant and *β*_2_ is significant in Model (3), it means that the impact of internal control quality on financial performance is completely achieved through the enterprise’s environmental protection investment transmission. If *β*_1_ is significant and *β*_2_ is significant, it indicates that the impact of internal control quality on financial performance is partially passed implementation of enterprise environmental protection investment transmission.

(2) $$\begin{array}{c} EPI=\alpha +\beta_{1}ICQ+\beta_{2}LEV+\beta_{3}ATO+\beta_{4}ID+\beta_{5}VOL+\beta_{6}TURN+\beta_{7}INSHARE\\ +\beta_{8}SIZE+\beta_{9}HHI+\sum YEAR\_Dummy+\varepsilon \end{array}$$

(3) $$\begin{array}{c} EPS=\alpha +\beta_{1}ICQ+\beta_{2}EPI+\beta_{3}LEV+\beta_{4}ATO+\beta_{5}ID+\beta_{6}VOL+\beta_{7}TURN\\ +\beta_{8}INSHARE+\beta_{9}SIZE+\beta_{10}HHI+\sum YEAR\_Dummy+\varepsilon \end{array}$$

In the above model, *α* is the model constant value, *β*_1_–*β*_10_ represent the coefficient values, and *ε* is the residual value.

## 4. Results

### 4.1. Descriptive Statistics

Table 2 provides the descriptive statistics of the variables. The difference between the maximum and minimum values of EPS is large, 13.0991 and 0.2415, respectively. The standard deviation of EPS is greater than its mean and median, indicating that the financial performances of the sample companies are quite different. The maximum and minimum values of EPI are 23.8949 and 6.5523, respectively, and the average is less than the median, indicating that the scale of environmental protection investment among the sample companies has large differences, and the scale of environmental protection investment of most sample companies is less than the average. The mean of ICQ is 6.4877, which is less than the median of 6.5157, indicating that the overall internal control quality of the sample enterprises is good.

### 4.2. Correlation Analysis

Table 3 shows that ICQ and EPS, ICQ and EPI, and EPI and EPS are positively correlated at a significance level of 1%, which initially verifies the previous hypothesis. In addition, the correlation coefficient between the two variables is less than 0.5, indicating that there is no serious multicollinearity problem. It was further tested by the VIF value.

### 4.3. Analysis of Regression Results

Table 4 provides the regression coefficient and results of each model. Firstly, from Model (1), ICQ and EPS are positively correlated at a 1% significance level, and the regression coefficient is 0.829, which verifies Hypothesis 1 (H1), indicating that. The higher is the internal control quality of the enterprise, the higher is the overall operating level of the enterprise, and the more conducive it is to improving corporate financial performance. In addition, it is also found that the change of the control variable CEO, the proportion of institutional investors, and the size of the enterprise are positively correlated with EPS at a 1% significance level, reflecting that the improvement of enterprise governance structure and the economies of scale can enhance the external competitiveness and promote the improvement of financial performance. LEV and HHI have negative correlations to financial performance at 1% and 5% significance levels, respectively, showing that excessively high debt levels and market competition tend to increase financial uncertainty, which increases the enterprise’s operating costs and inhibits financial performance level.

Secondly, in Model (2), ICQ and EPI are positively correlated at a 1% significance level, and the regression coefficient is 0.792, indicating that the higher is the quality of internal control, the more helpful it is for companies to embed environmental protection concepts in investment activities. It also suppresses the opportunistic behavior of managers’ investment, promotes the fulfillment of enterprise environmental responsibility, and expands the scale of enterprise environmental protection investment, which validates Hypothesis 2 (H2).

Similarly, from Model (3), we can see that there is a positive correlation between EPI and financial performance at a 1% significance level, and the regression coefficient is 0.013, which verifies Hypothesis 3 (H3). The above results indicate that enterprises actively investing in environmental protection can have a positive impact on financial performance. It also shows that the scale of enterprise environmental protection investment plays an intermediary role between internal control quality and financial performance. In addition, in Model (3), ICQ and EPS are also positively correlated at a 1% significance level, indicating that the scale of enterprise environmental protection investment plays a partial intermediary role in the positive impact of internal control on financial performance.

### 4.4. Robustness Tests

First, following Tang et al. [34], we used “enterprise’s newly added environmental protection investment scale/the average total assets” to measure the scale of enterprise environmental protection investment (EI). EPS has the possibility of indicator changes due to changes in the number of shares. Following Chen and Fan [8], we used cost expense profit ratio (CPR) to measure financial performance: CPR = total profit/(operating cost + sales expense + management expense + financial expense + business tax and surcharge). At the same time, CPR can reflect the profitability of the normal business activities of the enterprise. Second, we used “winsorize treatment of continuous variables with 1% and 99% quantile shrinkage” to minimize the disturbance of abnormal observations to this study. Third, we changed the model. To better solve the endogenous problems related to independent variables and unobservable individual effects, we used a fixed effect model for robustness testing.

The specific results are as follows in Table 5 and Table 6. There is no substantial change in the regression results, indicating that the research conclusion of this study is relatively reliable.

## 5. Discussion

### 5.1. Theoretical Contributions

The existing studies focus on the influencing factors of enterprise environmental protection investment [21,36,37,39] or the economic consequences [40,41,42,43] and do not use an intermediary variable for internal control capabilities to indirectly affect financial performance. Based on the perspective of resource-based perspective, this article regards corporate environmental protection investment activities as an important resource that is scarce, valuable, and difficult to replicate [44,45], which can positively affect corporate financial performance and effectively explain the motivation for companies to actively invest in environmental protection. Therefore, this study further expands the resource-based view theory from the perspective of resource acquisition. At the same time, it expands not only the study results of the relationship between internal control quality and enterprise environmental protection investment, but also the study results of the relationship between enterprise environmental protection investment and enterprise performance. Moreover, it provides a theoretical basis for the construction of enterprise social responsibility internal control system.

### 5.2. Managerial Contributions

This study clarified the intermediary role of corporate environmental protection investment in internal control quality and corporate performance, explored the way for enterprises to endogenously incorporate green development concepts and actively promoted environmental protection investment activities. It also provides evidence for enterprises to strengthen their internal environmental management capabilities and consciously assume environmental responsibility.

According to our study, enterprises should fully realize the importance of the construction and improvement of internal control and achieve sustainable development of enterprises by adopting voluntary environmental management actions. First, enterprises should regularly disclose detailed information about internal control to reduce the degree of information asymmetry between the enterprise and stakeholders, as well as between the enterprise owner and the operator. Second, enterprises should incorporate the environmental management performance assessment and punishment mechanism of the management into the internal control system to suppress the generation of agency costs and short-sighted behavior of the management, as well as maximize the effectiveness of the internal control supervision and incentive mechanism to ensure realization of long-term goals. Third, enterprises should correct the motivation for fulfilling environmental protection responsibilities, correctly understand the intermediary role of environmental protection investment in internal control quality and financial performance, and organically embed the environmental protection responsibilities of enterprises into the internal control processes. For example, enterprises should actively set up corresponding internal environmental protection departments, strengthen environmental protection and energy conservation publicity, provide environmental protection professional training for employees, create a good environmental protection corporate culture, etc. to promote the formation of the concept of green development from top to bottom. At the same time, enterprises should create a good green image and achieve enterprise value and sustainable development goals through environmental protection investment activities.

### 5.3. Policy Contributions

In the context of green development, the government has increasingly strict regulations on the external environment of enterprises, and the environmental management behavior of enterprises has been improved to a certain extent [46,47]. However, it has not been able to find an effective means to maximize the stimulation of enterprises to carry out environmental investment activities. At present, China’s heavy-polluting enterprises generally have insufficient investment in environmental protection [21]. Starting from the micro level, this article uses empirical evidence from companies in China’s A-share heavy-polluting industries to demonstrate the path for companies to achieve a win–win situation. On environmental issues, humanity is a community of destiny. China’s empirical evidence not only serves the construction of China’s ecological civilization, but also has practical significance for other countries to improve government environmental governance regulations and correctly guide enterprises’ active environmental practices.

According to our study, the government should pay attention to the effect of multiple co-governance on environmental governance, and guide enterprises to consciously fulfill their environmental protection responsibilities. First, the government should improve environmental laws and regulations, continue to strengthen the external environmental regulations of enterprises, and optimize market mechanisms such as the external business environment of enterprises. Second, the government should strengthen guidelines for the construction of internal control systems, further issue specific guidance documents, encourage and support third-party organizations to evaluate the effectiveness of internal control of enterprises, and create a good external environment.

## 6. Conclusions

This study investigated the actual implementation effect of the internal control system from a unique and important perspective (the level of enterprise environmental protection investment). It used empirical data to prove the role and importance of high-quality internal control in regulating enterprise environmental investment behaviors. The research conclusion provides a valuable supplement for the theoretical literature on the construction of internal control system of corporate social responsibility. In addition, this study proved that, in the process of strengthening internal management capacity building to achieve the “win–win” goal of environmental performance and economic performance, the enterprise’s environmental protection investment is an important intermediary transmission. The research conclusion enriches the resource-based view and provides evidence for enterprises to realize sustainable development through consciously undertaking environmental protection responsibility.

This study has certain limitations. First, this study only explored the mediating role of corporate environmental protection investment in the relationship between internal control and financial performance, without considering other adjustment factors. For example, companies with different property rights have differences in resource acquisition capabilities, risk preferences, and investment intentions. Therefore, future research can try to use the property rights as a moderating variable in the theoretical model of this research to explore whether it has an important moderating effect. Second, this study only used China’s A-share heavy-polluting companies as a sample, thus the conclusions of the study may not be fully applicable to all companies, and caution should be exercised in the promotion of the conclusions. Future studies can further select samples of non-heavy polluting industries to explore. Third, although the research data in this article are sufficient to support the research conclusions, the latest data can still be added to future research to further enhance the representativeness of the conclusions.

## Figures and Tables

**Table 1:** Variable description.

Type	Name	Code	Connotation
Explained variable	Financial performance	EPS	Net profit divided by total shares
Explanatory variable	Internal control quality	ICQ	Natural logarithm of internal control index
Intermediary variable	Environmental protection investment	EPI	Natural logarithm of newly added environmental protection investment
Control variable	Asset–liability ratio	LEV	Total liabilities divided by total assets
	Asset turnover ratio	ATO	Operating income divided by total assets
	The proportion of independent directors	ID	Number of independent directors divided by total number of boards
	Operating risk	VOL	Annual stock price volatility
	CEO change	TURN	If the CEO changed to 1 in the previous year, otherwise 0
	Institutional investor holdings	INSHARE	Institutional investor holdings divided by total shares
	Enterprise size	SIZE	Natural log of total assets
	Market the degree of competition	HHI	Hefindahl–Hirschman index
	Time	YEAR	Virtual variable

**Table 2:** Descriptive statistics.

Variables	N	Mean	Std	Maximum	Minimum	Median
EPS	2326	0.3771	0.6517	13.0991	−2.6767	0.2415
ICQ	2326	6.4877	0.1730	6.8797	4.1909	6.5157
EPI	2326	16.6052	2.4269	23.8949	6.5523	16.7236
LEV	2326	0.4855	0.2055	1.3518	0.0167	0.4979
ATO	2326	0.6522	0.3967	3.2808	0.0513	0.5714
ID	2326	0.3679	0.0537	0.7143	0.2308	0.3333
VOL	2326	42.2535	15.7877	133.3848	5.2898	39.2071
TURN	2326	0.8396	0.3670	1	0	1
INSHARE	2326	45.2132	22.7455	186.9690	0.0000	46.2644
SIZE	2326	22.6629	1.3333	28.5085	19.8377	22.5168
HHI	2326	0.1088	0.1229	0.9700	0.0153	0.0807

**Table 3:** Pearson’s correlation coefficients for the full sample.

Variables	EPS	ICQ	EPI	LEV	ATO	ID	VOL	TURN	INSHARE	SIZE	HHI
EPS	1										
ICQ	0.2872 ***	1									
EPI	0.0992 ***	0.1177 ***	1								
LEV	−0.2256 ***	−0.0509 **	0.2794 ***	1							
ATO	0.0691 ***	0.0770 ***	−0.1041 ***	−0.0365 *	1						
ID	0.0458 **	−0.0188	−0.0894 ***	−0.0536 ***	0.0601 ***	1					
VOL	−0.0416 **	−0.0296	−0.0905 ***	−0.0232	−0.0243	−0.0114	1				
TURN	0.0985 ***	0.0486 **	−0.0061	−0.1196 ***	−0.0222	−0.0203	0.0049	1			
INSHARE	0.1272 ***	0.1082 ***	0.1421 ***	0.1679 ***	0.1209 ***	0.0021	−0.0805 ***	−0.0896 ***	1		
SIZE	0.1498 ***	0.1576 ***	0.4971 ***	0.4739 ***	−0.0345 *	0.0083	−0.2149 ***	−0.0658 ***	0.4170 ***	1	
HHI	−0.0201	0.0128	−0.0995 ***	−0.0307	0.1273 ***	0.0743 ***	−0.0542 ***	0.0169	0.0208	−0.0448 **	1

Note: *, **, *** indicate significance at the 10%, 5%, and 1% levels, respectively, and the same below.

**Table 4:** Regression coefficient and results of each model.

Variables	Model (1)	Model (2)	Model (3)
	EPS	EPI	EPS
ICQ	0.829 ***	0.792 ***	0.819 ***
	(7.94)	(3.03)	(7.89)
EPI			0.013 ***
			(2.68)
LEV	−1.062 ***	0.643 **	−1.070 ***
	(−10.57)	(2.49)	(−10.62)
ATO	0.052 *	−0.435 ***	0.058 *
	(1.72)	(−3.73)	(1.90)
ID	0.374	−3.629 ***	0.421
	(1.23)	(−4.51)	(1.38)
VOL	0.003 *	0.004	0.003 *
	(1.93)	(1.00)	(1.89)
TURN	0.119 ***	0.141	0.118 ***
	(3.90)	(1.19)	(3.86)
INSHARE	0.002 ***	−0.007 ***	0.002 ***
	(3.25)	(−3.11)	(3.38)
SIZE	0.132 ***	0.894 ***	0.121 ***
	(8.35)	(19.98)	(7.70)
HHI	−0.181 **	−1.245 ***	−0.165 **
	(−2.32)	(−3.45)	(−2.14)
YEAR	Control	Control	Control
Constant	−7.777 ***	−7.302 ***	−7.683 ***
	(−10.79)	(−4.15)	(−10.75)
N	2326	2326	2326
R^2^	0.214	0.277	0.216
R^2^_Adj	0.208	0.271	0.209
F	17.36 ***	47.03 ***	16.52 ***
VIF_Max	1.92	1.92	2.21

Note: *, **, *** indicate significance at the 10%, 5%, and 1% levels, respectively.

**Table 5:** Robustness test (Alternative measures).

Variables	EI			CPR		
	Model (1)	Model (2)	Model (3)	Model (1)	Model (2)	Model (3)
	EPS	EI	EPS	CPR	EI	CPR
ICQ	0.795 ***	0.015 ***	0.780 ***	0.169 ***	0.015 ***	0.164 ***
	(7.42)	(3.85)	(7.33)	(5.72)	(3.85)	(5.54)
EI			1.037 ***			0.345 *
			(4.52)			(1.84)
LEV	−1.057 ***	0.028 ***	−1.086 ***	−0.385 ***	0.028 ***	−0.394 ***
	(−10.09)	(2.85)	(−10.38)	(−10.10)	(2.85)	(−10.78)
ATO	0.060 *	−0.012 ***	0.073 **	−0.091 ***	−0.012 ***	−0.087 ***
	(1.88)	(−5.47)	(2.25)	(−7.26)	(−5.47)	(−6.93)
ID	0.443	−0.012	0.455	0.162 *	−0.012	0.166 *
	(1.38)	(−0.67)	(1.42)	(1.80)	(−0.67)	(1.85)
VOL	0.002	0.000	0.002	−0.000	0.000	−0.000
	(1.34)	(1.03)	(1.28)	(−0.98)	(1.03)	(−1.06)
TURN	0.135 ***	0.003	0.132 ***	0.016	0.003	0.015
	(4.34)	(1.29)	(4.27)	(1.16)	(1.29)	(1.09)
INSHARE	0.002 ***	0.000	0.002 ***	0.000 **	0.000	0.000 **
	(2.97)	(0.28)	(2.96)	(2.19)	(0.28)	(2.17)
SIZE	0.138 ***	−0.004 **	0.142 ***	0.029 ***	−0.004 **	0.031 ***
	(8.16)	(−2.49)	(8.41)	(5.69)	(−2.49)	(6.10)
HHI	−0.170 **	−0.002	−0.168 **	0.022	−0.002	0.023
	(−2.18)	(−0.22)	(−2.18)	(0.70)	(−0.22)	(0.76)
YEAR	Control	Control	Control	Control	Control	Control
Constant	−7.734 ***	−0.009	−7.725 ***	−1.474 ***	−0.009	−1.471 ***
	(−10.39)	(−0.27)	(−10.44)	(−6.90)	(−0.27)	(−6.96)
N	2089	2089	2089	2089	2089	2089
R^2^	0.218	0.038	0.221	0.191	0.038	0.195
R^2^_Adj	0.211	0.0295	0.214	0.184	0.0295	0.188
F	15.94 ***	5.188 ***	15.84 ***	14.56 ***	5.188 ***	15.69 ***

Note: *, **, *** indicate significance at the 10%, 5%, and 1% levels, respectively.

**Table 6:** Robustness test (Winsorize treatment and fixed effect model).

	Winsorized			Fixed Effect Model		
	Model (1)	Model (2)	Model (3)	Model (1)	Model (2)	Model (3)
	EPS	EI	EPS	EPS	EI	EPS
ICQ	1.066 ***	0.018 ***	1.046 ***	0.813 ***	0.020 ***	0.785 ***
	(11.05)	(4.89)	(10.88)	(10.80)	(4.28)	(10.40)
EI			1.087 ***			1.411 ***
			(3.22)			(3.46)
LEV	−0.903 ***	0.017 ***	−0.921 ***	−0.960 ***	0.015 **	−0.981 ***
	(−13.02)	(4.00)	(−13.39)	(−9.26)	(2.22)	(−9.48)
ATO	0.052 *	−0.011 ***	0.064 **	0.400 ***	−0.009 ***	0.413 ***
	(1.80)	(−7.10)	(2.17)	(7.22)	(−2.58)	(7.46)
ID	0.266	−0.020	0.287	−0.755 ***	0.010	−0.770 ***
	(1.41)	(−1.52)	(1.52)	(−2.76)	(0.61)	(−2.82)
VOL	0.002 **	0.000	0.002 **	−0.000	−0.000	0.000
	(2.50)	(1.63)	(2.34)	(−0.05)	(−0.60)	(0.00)
TURN	0.108 ***	0.002	0.106 ***	0.045 *	0.003 **	0.040
	(3.89)	(1.04)	(3.84)	(1.79)	(2.04)	(1.61)
INSHARE	0.001 ***	−0.000	0.001 ***	0.000	−0.000	0.000
	(2.87)	(−0.25)	(2.89)	(0.29)	(−0.94)	(0.38)
SIZE	0.111 ***	−0.002 ***	0.113 ***	0.182 ***	−0.002	0.185 ***
	(9.30)	(−2.84)	(9.57)	(7.59)	(−1.28)	(7.73)
HHI	−0.166 **	−0.007	−0.158 **	0.187	−0.012	0.205
	(−2.36)	(−1.44)	(−2.27)	(0.88)	(−0.91)	(0.96)
YEAR	Control	Control	Control	-	-	-
Constant	−8.873 ***	−0.058 **	−8.811 ***	−8.623 ***	−0.078	−8.513 ***
	(−14.45)	(−2.34)	(−14.38)	(−11.07)	(−1.58)	(−10.96)
N	2089	2089	2089	2089	2089	2089
R^2^	0.270	0.045	0.273	0.176	0.022	0.183
R^2^_Adj	0.264	0.0372	0.267	-	-	-
F	25.83 ***	6.074 ***	25.33 ***	35.75 ***	3.668 ***	33.61 ***

Note: *, **, *** indicate significance at the 10%, 5%, and 1% levels, respectively.
